# *In situ* probing of phase transition kinetics of single CsPbI_3_ perovskite quantum dots *via* nano-impact electrochemistry

**DOI:** 10.1039/d6sc04719j

**Published:** 2026-08-06

**Authors:** Xiaolin Zhang, Yu Tian, Yuanhang Zhu, Nanlin Zhang, Chin-Wen Chen, Xiuting Li

**Affiliations:** a Institute for Advanced Study, Shenzhen University Shenzhen 518060 China xiuting.li@szu.edu.cn; b YangMing Quantum Technology Ltd Shenzhen 518000 China; c Department of Molecular Science and Engineering, National Taipei University of Technology Taipei 10608 Taiwan

## Abstract

Purification using polar solvents such as ethyl acetate (EtOAc) inevitably triggers a γ-to-δ phase transition in perovskite quantum dots (QDs), thereby deteriorating their structural integrity and optoelectronic performance. Understanding the intrinsic kinetics of this phase transition at the single-particle level remains a big challenge. Herein, we report an *in situ* electrochemical strategy based on nano-impact electrochemistry to probe the phase transition kinetics of CsPbI_3_ QDs in EtOAc at the single-nanoparticle level. By exploiting the distinct Pb reduction overpotentials of γ- and δ-phase CsPbI_3_, the γ-phase fraction of single QDs during solvent exposure was quantified in real time through statistical analysis of current spikes generated as individual QDs collided with a microelectrode. Kinetic modeling using the Johnson–Mehl–Avrami–Kolmogorov framework suggests that the γ-to-δ phase transition proceeds *via* heterogeneous nucleation followed by low-dimensional interfacial propagation under strong nanoscale confinement. Compared with ensemble-averaged *ex situ* XRD measurements, the nano-impact approach enables more accurate and precise kinetic fitting and improved mechanistic resolution of the phase-transition process. This work demonstrates that nano-impact electrochemistry enables *in situ* monitoring of phase transition kinetics at the single-particle level, revealing solvent-induced structural evolution and guiding the design of more robust purification strategies to stabilize perovskite QDs.

## Abstract 2

Nano-impact electrochemistry enables *in situ* minitoring of the γ-to-δ phase transition kinetics of CsPbI_3_ QDs in EtOAc at the single-nanoparticle level.

## Graphical abstract

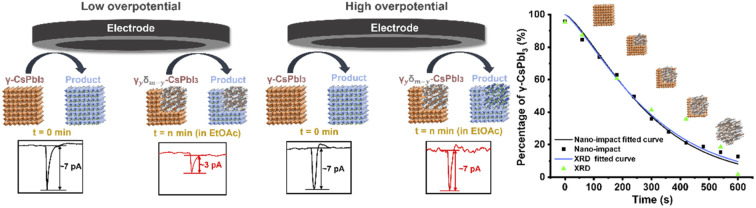

## Introduction

As a member of all-inorganic perovskites, CsPbI_3_ has shown superior potential in photovoltaic and optoelectronic applications^1–4^ because of its outstanding properties, including appropriate bandgaps,^5^ fast carrier mobility, high absorption coefficient and excellent thermal stability.^6^ However, since the ionic radius of Cs^+^ is too small to accommodate the octahedron [PbI_6_]^4−^, its luminous black phases (α, β and γ) can easily transform into the non-luminous yellow phase (δ), which significantly limits its further development. The preparation of CsPbI_3_ into nanoscale quantum dots (QDs) is one of the most effective ways to stabilize the structure because of the high surface energy and organic ligands of QDs which can improve the phase transition energy barrier.^7^ But excess organic ligands need to be removed by using polar solvents *e.g.* ethyl acetate (EtOAc) after synthesis to improve charge delocalization and carrier transport^8^ and to help QDs fully precipitate.^9^ During this purification process, defects are generated and then lead to surface reconstruction or even phase transitions.^10,11^ As a result, *in situ* determining and understanding the kinetics of phase transition during purification is vital to optimize the purification strategy and obtain high quality QDs.

To date, however, *in situ* detection of the phase transition kinetics of CsPbI_3_ QDs in solution, especially at the single-particle level, undoubtedly challenges the existing techniques. *In situ* X-ray diffraction (XRD) is primarily suitable for solid-state samples and, in some cases, highly concentrated solutions.^12,13^ In liquid-phase systems, substantial background noise, diminished diffraction intensity, and the inherent Brownian motion of nanoparticles make accurate *in situ* measurements both difficult and unreliable.^14^ Furthermore, only average behavior from ensembles can be detected with this technique, which limits its ability to reveal the intrinsic properties of individual particles. Although *in situ* electron microscopy can probe single entities, it is costly and high-energy electron beams have been reported to adversely affect perovskite QDs.^15^

Nano-impact electrochemistry—an emerging technique in which electrochemical signals arise from individual nanoparticles colliding with a microelectrode and undergoing processes such as faradaic electrolysis, electrocatalytic amplification,^16^ or diffusion blocking—has become a powerful *in situ* method for detecting particle concentration, size, and charge-transfer kinetics and for real-time monitoring of nanoparticle agglomeration/aggregation kinetics in solution.^17^ For instance, Zhang's group used current lifetimes to distinguish Au nanoparticle sizes and to track their time-dependent agglomeration/aggregation behaviors.^18^ Our previous work also demonstrated the aging process of Ag nanoparticles in real world media using the nano-impact technique.^19^ However, more complex processes such as phase transitions have not yet been probed on the single nanoparticle level, which limits the development of kinetic models and the rational design of nanoparticles.

Herein, we develop an *in situ* strategy for probing the phase transition kinetics of CsPbI_3_ QDs in EtOAc at the single nanoparticle level by using nano-impact electrochemistry. As demonstrated in our previous work, the Pb reduction in γ-phase QD ensembles occurs at lower overpotentials than that in δ-phase so that the phase transition level can be electrochemically determined.^20^ In this work, the current spikes generated by individual QDs exposed to EtOAc were *in situ* detected at different overpotentials with both cyclic voltammetry (CV) and chronoamperometry (CA). The fraction of γ-CsPbI_3_ within individual nanoparticles during purification was analyzed and hence the kinetics of the phase transition of CsPbI_3_ QDs in polar solvents was revealed. As a well-established and widely adopted kinetic model for describing phase transitions involving nucleation and growth, the Johnson–Mehl–Avrami–Kolmogorov (JMAK) model was used to conduct the kinetic analysis and it is evidenced that the solvent-induced γ-to-δ phase transition proceeds through heterogeneous nucleation followed by low-dimensional interfacial propagation under nanoscale confinement. The nano-impact approach enables more accurate and precise kinetic analysis with enhanced mechanistic resolution than ensemble-averaged *ex situ* XRD measurements. The mechanistic insights obtained in this work highlight the importance of suppressing surface nucleation and retarding interfacial propagation during purification, thereby providing a rational basis for designing more robust purification strategies for perovskite QDs.

## Results and discussion

Three different types of electrochemical measurement were attempted to determine the phase transition level of CsPbI_3_ QDs in purification: the QD modified glassy carbon (GC) electrode, the QD adsorbed carbon fiber (CF) microelectrode and the nano-impact electrochemistry. First the influence of electrolyte was investigated, and its concentration was optimized by applying *ex situ* CV with the modified GC electrode and modified microelectrode. Second, the nano-impact electrochemistry was conducted at low concentration of electrolyte through CA to *ex situ* determine the phase transition level of the purified CsPbI_3_ QDs. Next, nano-impact experiments were further performed using CV and CA to investigate the *in situ* phase transition kinetics of CsPbI_3_ QDs during purification. CV enabled the qualitative tracking of phase evolution through the time-dependent collision frequency of individual QDs at different overpotentials, while CA provided quantitative kinetic information by recording the spike charge in real time. Finally, the results were compared with those of *ex situ* XRD analysis, and the phase transition kinetics was clarified.

**Fig. 1:**
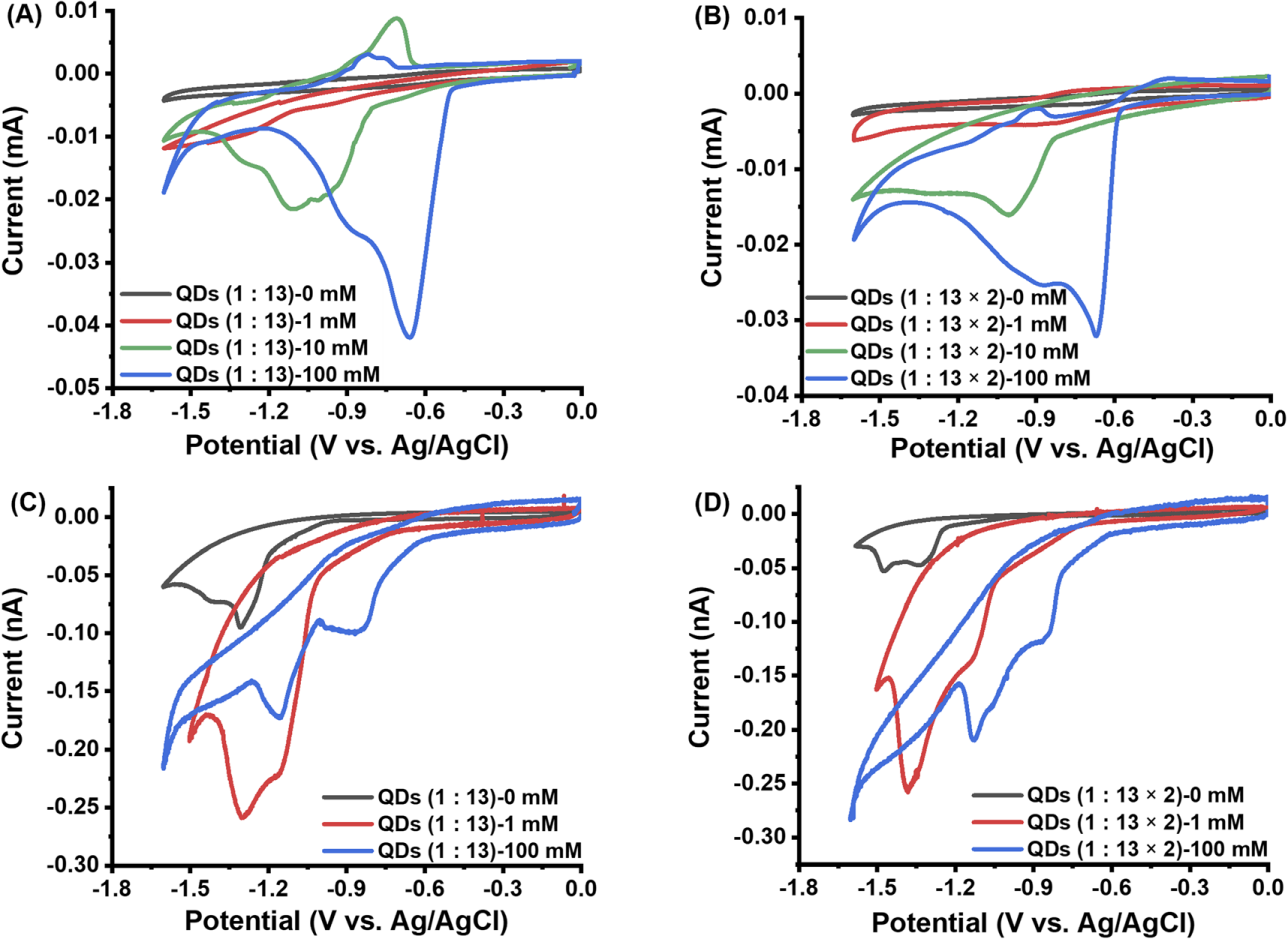
Cyclic voltammograms of a GC electrode dropcast with about 30 µg CsPbI_3_ QDs (1 : 13) (A) and CsPbI_3_ QDs (1 : 13 × 2) (B) in 0 mM, 1 mM and 100 mM electrolyte. CV of a CF microelectrode absorbed with CsPbI_3_ QDs (1 : 13) (C) and CsPbI_3_ QDs (1 : 13 × 2) (D) (sticking in their suspensions for 5 s) in 0 mM, 1 mM and 100 mM electrolyte.

Given that electrolyte might influence the stability of QD solution, the concentration of electrolyte needs to be first optimized to minimize its influence and to provide enough supporting electrolyte for electrochemical reactions. In our previous work, distinguishable reduction peaks for the two different phases have been observed in the purified CsPbI_3_ QDs. Herein, the purified CsPbI_3_ QDs with two different levels of phase transition, CsPbI_3_ QDs (1 : 13) and CsPbI_3_ QDs (1 : 13 × 2), were employed to investigate the effect of electrolyte concentration on the CV behavior of two phases. The purified QDs were named according to the volume ratio of the QD solution and polar solvent used in the purification process. Specifically, CsPbI_3_ QDs (1 : 13 × 2) were obtained by subjecting 3 mL of QD solution to two successive purification cycles with 39 mL of EtOAc in each cycle. Details about the preparation and purification processes can be found in the Experimental section in the SI (SI Section 1). Fig. 1A and B shows the cyclic voltammograms of a GC macroelectrode dropcast with the CsPbI_3_ QDs (1 : 13) and CsPbI_3_ QDs (1 : 13 × 2) in dichloromethane containing 100 mM, 10 mM, 1 mM and 0 mM Bu_4_NPF_6_. In Fig. 1A, two distinguishable reduction peaks from the CsPbI_3_ QDs (1 : 13) were observed at −0.66 V and −0.89 V *vs.* Ag/AgCl in 100 mM electrolyte, which are attributed to the reduction of Pb(ii) in γ-CsPbI_3_ and δ-CsPbI_3_ respectively.^20^ γ-CsPbI_3_ is reduced more easily because the conduction band minimum (CBM) of CsPbI_3_ is mainly composed of Pb 6p–I 5p antibonding states and mainly derived from the unoccupied Pb p orbitals. It has been reported that DFT calculations demonstrated a much lower CBM of the γ-phase CsPbI_3_ than that of the δ-phase CsPbI_3_.^21^ It can be found in Fig. 1A that lower electrolyte concentrations lead to significant distortion and even disappearance of reduction peaks. The IR drop is responsible for the decreased current which might even be covered by the capacitive current of the macroelectrode.^22^ However, although 100 mM electrolyte is supportive, the photoluminescence (PL) stability of CsPbI_3_ QDs shows that the PL intensity of QDs drops around 10% after 2 min in 100 mM electrolyte (Fig. S1A), implying that the macroelectrode measurement is not applicable to investigate the *in situ* phase transition during EtOAc purification. The phenomenon of CsPbI_3_ QDs (1 : 13 × 2) is similar to that of CsPbI_3_ QDs (1 : 13) except for the higher current at high overpotentials from the increased δ-CsPbI_3_ content of CsPbI_3_ QDs (1 : 13 × 2) under purification with more anti-solvent.

CV of the CF microelectrode adsorbed separately with CsPbI_3_ QDs (1 : 13) and CsPbI_3_ QDs (1 : 13 × 2) was further conducted in solution with different concentrations of electrolyte (Fig. 1C and D). The reduction peaks are clearly observed in 100 mM and 1 mM and even in the absence of electrolyte and much less distortion occurs in low electrolyte concentrations than that observed in the modified GC electrode, indicating that smaller electrolyte concentration is needed for microelectrode measurements. The PL intensity of CsPbI_3_ QDs almost remains constant in the first 2 min and drops less than 10% after 6 min in 1 mM electrolyte (Fig. S1C), which means that low electrolyte concentration has slight influence on CsPbI_3_ QDs.

In 100 mM electrolyte, two reduction peaks at the CF microelectrode occur at −0.83 V and −1.15 V respectively, lower than the peak potentials of the modified GC electrode. It is likely due to the loose contact between the adsorbed nanoparticles and microelectrode surface.^23^ This is similar to the nano-impact measurements where higher overpotentials are required for single particle electrolysis.^24^ The relevant peak potentials are summarized in Table 1. It is further found that in 100 mM electrolyte the peak potential difference (Δ*E* = *E*_peak1_ − *E*_peak2_) at the microelectrode is 0.32 V, larger than that at the GC electrode (0.23 V); this is because the irregular film formed on the GC electrode surface associated with the dropcast technique easily leads to the merging of two peaks. Besides, for both the modified GC electrode and CF microelectrode, Δ*E* decreases as the electrolyte concentration drops from 100 mV to 0 mV, which means that the reduction potential of γ-CsPbI_3_ is more significantly affected by the decrease in electrolyte concentration than that of δ-CsPbI_3_. This is likely due to the higher reductive faradaic current of γ-CsPbI_3_ than that of δ-CsPbI_3_, which leads to a larger IR drop. The absolute reduction currents from each phase can be more clearly reflected by CV of the dropcast GC (Fig. 1A and B). The reduction current of CsPbI_3_ at the CF microelectrode is difficult to be determined due to the influence of background current from solvent breakdown especially in high concentration electrolyte (Fig. S2). Overall, it has been evidenced that the CV of QDs adsorbed at the CF microelectrode at low electrolyte concentration has potential to be used to estimate the phase transition level of QDs with careful background correction and peak separation. However, these ways of data analysis likely cause certain inaccuracy issues in the quantification results, which, however, are expected to be eliminated by conducting single nanoparticle detection *via* nano-impact experiments.

**Table 1:** Reduction peak potentials recorded from the CV curves in Fig. 1A and C

Working electrode	Electrolyte concentration (mM)	*E*_peak1_ (V)	*E*_peak2_ (V)	Δ*E*(*E*_peak1_ − *E*_peak2_) (V)
GC electrode	100	−0.66	−0.89	0.23
	10	−1.08	−1.30	0.22
CF electrode	100	−0.83	−1.15	0.32
	1	−1.15	−1.34	0.19
	0	−1.31	−1.43	0.12

**Fig. 2:**
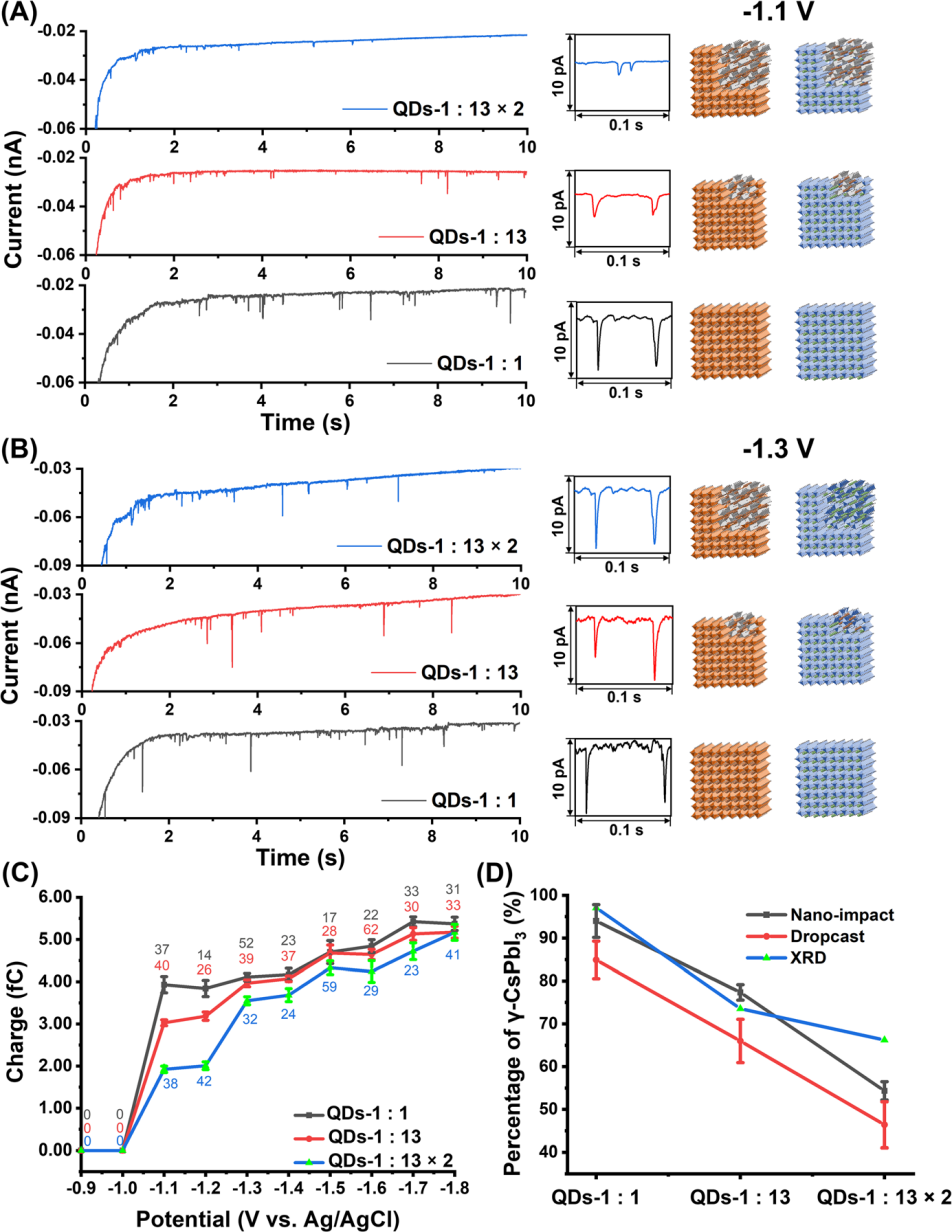
Chronoamperograms of CsPbI_3_ QDs (1 : 1), CsPbI_3_ QDs (1 : 13) and CsPbI_3_ QDs (1 : 13 × 2) at −1.1 V (A) and −1.3 V (B) in the presence of 1 mM electrolyte. Charge statistics of spikes obtained at different potentials, with the numbers indicating the corresponding impact counts (C). Percentage of γ-CsPbI_3_ obtained by different methods (D).

In the nano-impact experiments, CA was first conducted to quantify the phase transition extent of individual CsPbI_3_ QDs (1 : 1), CsPbI_3_ QDs (1 : 13) and CsPbI_3_ QDs (1 : 13 × 2) in 1 mM electrolyte solution. CsPbI_3_ QDs were added to the electrolyte following which the microelectrode was applied with reductive potentials. Fig. S3A–C shows the current–time transients recorded from −0.9 V to −1.8 V *vs.* Ag/AgCl for the three samples. It is found that current spikes appear in the potential range of −1.1 V to −1.8 V whilst no current spikes are observed at −0.9 V and −1.0 V, indicating that reduction of single CsPbI_3_ QDs starts at around −1.1 V. The larger overpotential than that of the ensembles is probably associated with the loose contact of single QDs with the microelectrode.^23^Fig. 2A and B show the CA of the three CsPbI_3_ QD samples at −1.1 V and −1.3 V respectively. As shown in the insets of Fig. 2A, the sample with a higher degree of purification exhibits smaller current spikes at −1.1 V, which indicates that only γ-CsPbI_3_ was reduced at −1.1 V and the content of γ-CsPbI_3_ decreases as purification becomes severe. In contrast, the impact signals generated by the collision of CsPbI_3_ QDs (1 : 1), CsPbI_3_ QDs (1 : 13) and CsPbI_3_ QDs (1 : 13 × 2) at −1.3 V are almost the same and larger than those at −1.1 V (Fig. 2B), indicating that Pb(ii) in both γ-CsPbI_3_ and δ-CsPbI_3_ was reduced at −1.3 V. Therefore, nano-impact experiments enabled the quantitative determination of phase transition levels in individual CsPbI_3_ QDs by exploiting the distinct Pb reduction potentials of γ- and δ-phases, allowing the γ-phase fraction to be directly extracted from spike charges at different overpotentials.

The charge transferred during single particle collision events was obtained by integrating the current transient spikes and the results from −0.9 V to −1.8 V are shown in Fig. 2C. For CsPbI_3_ QDs (1 : 1), only one charge step is observed at −1.1 V, and the charge is 3.9 ± 0.2 fC; this is slightly higher than the theoretical charge of 3 ± 1 fC calculated for the complete reduction of a CsPbI_3_ QD with 13 ± 2 nm in diameter. This is likely due to the slight aggregation/agglomeration of QDs during complex *ex situ* purification which includes multiple steps of centrifugation and freezing. The results imply that the reduction of γ-CsPbI_3_ starts at −1.1 V and the CsPbI_3_ QDs (1 : 1) mainly consist of γ-CsPbI_3_. There is almost no phase transition for this sample. For CsPbI_3_ QDs (1 : 13) and CsPbI_3_ QDs (1 : 13 × 2), the first step appears at −1.1 V similar to that for CsPbI_3_ QDs (1 : 1) but much smaller charge is obtained. Specifically, the first charge steps of CsPbI_3_ QDs (1 : 13) and CsPbI_3_ QDs (1 : 13 × 2) are 3.03 ± 0.07 fC and 1.92 ± 0.08 fC, respectively. This means that γ-CsPbI_3_ contents in these two samples, especially in CsPbI_3_ QDs (1 : 13 × 2), are lower than those in CsPbI_3_ QDs (1 : 1) due to the phase transition caused by purification.

The second step appears at −1.3 V in CsPbI_3_ QDs (1 : 13) and CsPbI_3_ QDs (1 : 13 × 2). The charge of CsPbI_3_ QDs (1 : 13) and CsPbI_3_ QDs (1 : 13 × 2) increased from 3.03 ± 0.07 fC to 3.96 ± 0.08 fC and from 1.92 ± 0.08 fC to 3.55 ± 0.09 fC, respectively. This indicates that the reduction of δ-CsPbI_3_ occurs at about −1.3 V and the phase transition degree of CsPbI_3_ QDs (1 : 1), CsPbI_3_ QDs (1 : 13) and CsPbI_3_ QDs (1 : 13 × 2) increases successively. It can be further found that the charges at −1.3 V and −1.4 V of the three samples are close, reflecting the reduction of both phases of QDs at these potentials. Note that when the applied potential is higher than −1.4 V, the oxygen reduction reaction catalyzed by CsPbI_3_ QDs may occur,^25^ resulting in a further increase in the charge of the spikes.

By analyzing the spikes recorded in the range from −1.1 V to −1.4 V, the percentage of γ-CsPbI_3_ was calculated using the following formula: 
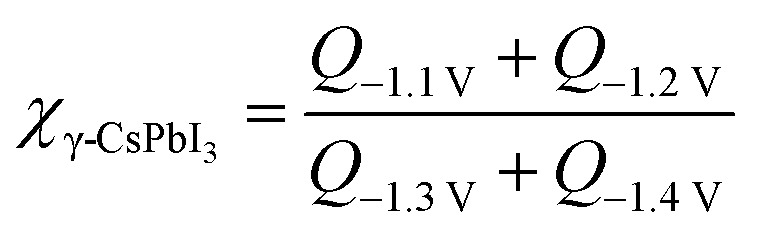
 and compared with the result obtained from XRD (Fig. S4A and Table S1) and from the dropcast experiments in our previous work (Fig. S4B).^20^ As shown in Fig. 2D, three different experimental methods all demonstrate that as more anti-solvent is used, more γ-CsPbI_3_ is converted into δ-CsPbI_3_. The percentage of γ-CsPbI_3_ obtained by the nano-impact experiments was close to that of XRD and about 10% higher than that of the dropcast experiments. The lower γ-phase content from the dropcast experiments likely results from the influence of electrolyte and/or the error caused by peak separation. Therefore, the results from nano-impact experiments are more accurate than those of the ensemble measurements. Specifically, the γ-phase contents in CsPbI_3_ QDs (1 : 1), CsPbI_3_ QDs (1 : 13) and CsPbI_3_ QDs (1 : 13 × 2) are 94.0%, 77.4% and 54.3%, respectively.

**Fig. 3:**
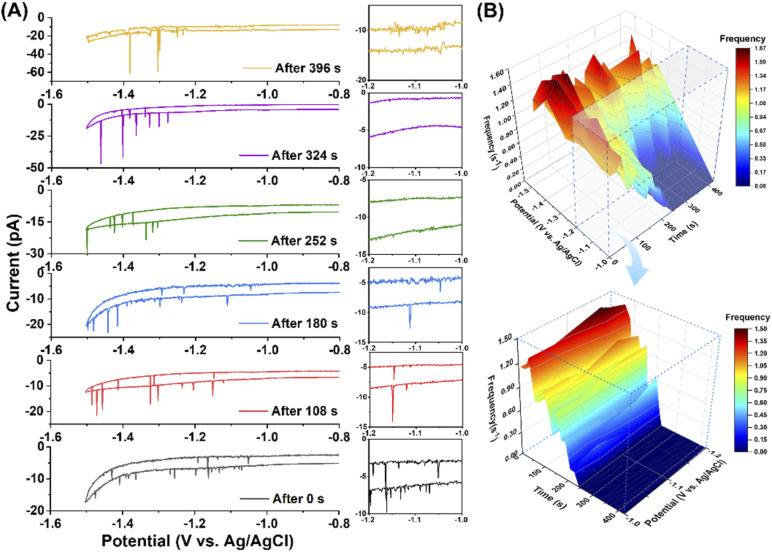
Cyclic voltammograms of unpurified CsPbI_3_ QDs in 1 mM electrolyte (A). 3D maps illustrating the plot of impact frequency against time from −1.0 V to −1.5 V (upper) and the zoom-in maps from −1.0 V to −1.2 V (lower) (B).

Having evidenced the ability of nano-impact methodology in *ex situ* determining the phase transition level of the QDs after purification, the nano-impact method which also enables the *in situ* and real-time determination was further employed to investigate the phase transition kinetics of QDs during the purification process. This is realized by first performing nano-impact experiments with CV in a suspension containing 20 µL EtOAc, 6 µL unpurified QD sample and 1 mM electrolyte. Continuous CV scans were intermediately recorded after the EtOAc and QDs were introduced into the electrolyte. The first six scans were plotted and are shown in Fig. 3A. Spikes appear at potential lower than −1.0 V, which is consistent with the CA results in *ex situ* nano-impact experiments and associated with the reduction of γ-CsPbI_3_. The average spike charge is lower than that of the theoretical value; this is probably associated with the limited temporal resolution of CV (sampling rate: 50 Hz) compared with that of CA (5000 Hz). This leads to incomplete recordings of the current spikes on CV and hence the integrated charge is underestimated. Therefore, CV is mainly used to probe phase transitions by measuring the frequency of collision at different overpotentials. It is found that, as time goes on, the spike frequency decreases significantly at low overpotentials but remains similar at high overpotentials. To clarify the relationship between the spike frequency, time, and potential, the relevant 3D maps were drawn and are shown in Fig. 3B. It is found that the frequency of the spikes at −1.0 V to −1.2 V gradually decreased from around 1.2 ± 0.1 s^−1^ to 0 s^−1^ with time (as shown in the zoomed-in lower figure), while the frequency of spikes at high overpotentials from −1.3 V to −1.5 V maintained at about 1.3 ± 0.1 s^−1^. It is proved that γ-CsPbI_3_ became less as it was gradually converted to δ-CsPbI_3_ in polar solvent. After about 250 s, almost no spikes were detected at potential less than −1.2 V, which is shorter than the time (600 s) for 90% phase transition from the CA results. This is likely because of the higher background noise of CV (∼0.6 pA) than that of CA (∼0.2 pA), which covers the small current spikes from the QDs with a severe phase transition at low overpotential in CV. This also explains that the spike frequency in CV is lower than that in CA. Moreover, the frequency in CV is found to be independent of the exposure time at high overpotentials, indicating that the QDs with different phase compositions have similar collision behavior.

The nano-impact experiment in the absence of electrolyte was also performed. Current spikes appear at over −1.2 V, slightly later than that in 1 mM electrolyte due to *IR* drop. The spikes appearing in a low overpotential range of −1.2 V to −1.4 V disappear after about 280 s (Fig. S5). This phenomenon agrees well with that in 1 mM electrolyte. However, the whole impact frequency of the electrolyte-free system was ∼0.2 s^−1^, lower than that in the 1 mM electrolyte solution because of the barrier of charge transport.^26^

**Fig. 4:**
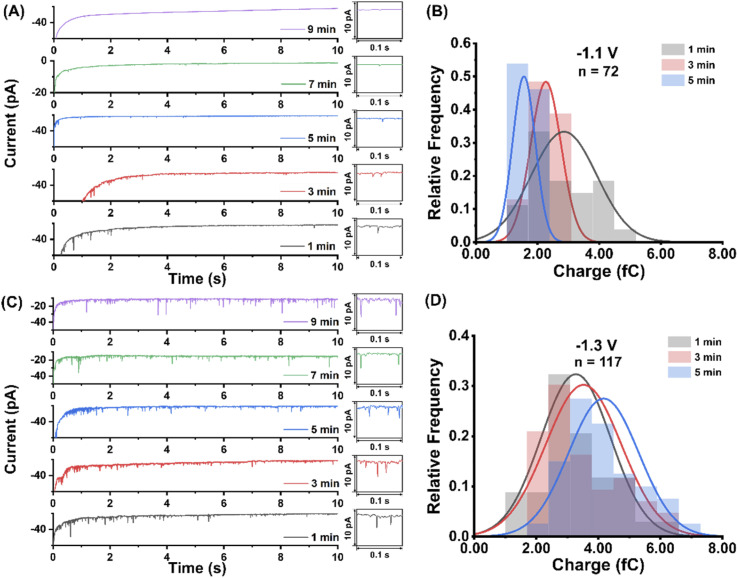
Chronoamperograms of unpurified CsPbI_3_ QDs exposed to EtOAc for different times at −1.1 V (A) and −1.3 V (C). The corresponding relative frequency distributions of the spike charge at −1.1 V for 72 impacts (B) and −1.3 V for 117 impacts (D).

CV probed the *in situ* phase transition of single CsPbI_3_ QDs in the presence of polar solvent EtOAc and indicated the reduction potential windows of the two phases. To collect more accurate and enough statistical data for quantitative analysis, CA was further employed in nano-impact experiments, in which continuous scans were recorded intermediately once the unpurified CsPbI_3_ QDs were mixed with polar solvent (Fig. S6A–D). CAs of CsPbI_3_ QDs exposed to EtOAc in 1 mM electrolyte for 1 min, 3 min, 5 min, 7 min and 9 min recorded at −1.1 V and −1.3 V are shown in Fig. 4A and C respectively. It is seen that the spike charge at −1.1 V in the first 5 min becomes smaller with the increase in the mixing time with EtOAc (Fig. 4B), while the spike charge at −1.3 V remains the same from 1 min to 5 min (Fig. 4D). This suggests that increasing mixing time with EtOAc leads to a higher phase transition degree of QDs within 5 min. However, the spikes at −1.1 V after 5 min decrease slower than before and the spike current at −1.3 V increases rapidly from 5 min to 9 min. This is probably because CsPbI_3_ QDs undergo phase transitions and aggregation at the same time after 5 min.

**Fig. 5:**
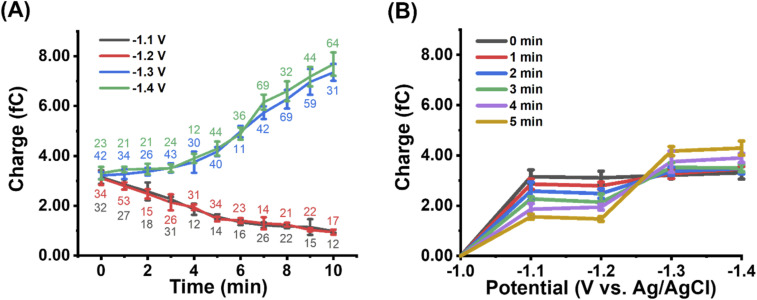
Charge statistics of spikes obtained at different exposure times and potentials, with the numbers indicating the corresponding impact counts (A). The spike charge in the first 5 min at different potentials (B).

The spike charges from 0 min to 10 min at different potentials of CA were analyzed and are shown in Fig. 5A. It is obvious that the drop rate of the spikes charges at low potentials (−1.1 V and −1.2 V) became slow after 5 min. Besides, the charge of spikes at high potentials (−1.3 V and −1.4 V) is almost unchanged in the first 5 min, whist after that, *Q*_−1.3 V_ and *Q*_−1.4 V_ rapidly increased to 7.4 ± 0.3 fC and 7.7 ± 0.5 fC because of particle aggregation. For clarity, the charge in the first 5 min at different potentials is extracted and plotted (Fig. 5B). *Q*_−1.1 V_ and *Q*_−1.2 V_ at 0 min are 3.2 ± 0.3 fC and 3.1 ± 0.3 fC respectively, which are very close to the complete Pb reduction of 3 ± 1 fC. It is found that both *Q*_−1.1 V_ and *Q*_−1.2 V_ gradually decreased to 1.6 ± 0.1 fC and 1.5 ± 0.1 fC from 0 min to 5 min due to the phase transition. Note that the charge of spikes after 5 min is not from single QD collision anymore since aggregation occurs. However, phase composition can still be quantified because the QD aggregates are expected to show the same phase transition level as the individual QDs.

**Fig. 6:**
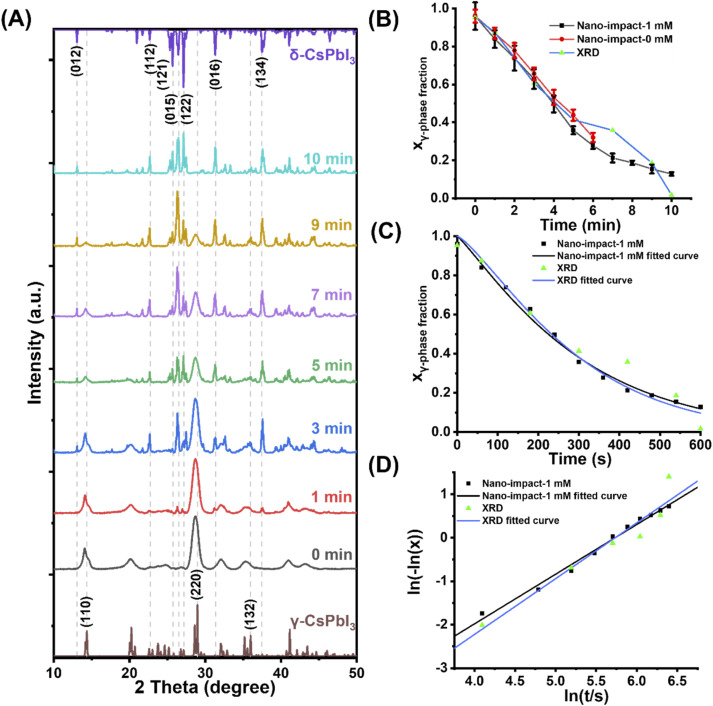
*Ex situ* XRD of CsPbI_3_ QDs exposed to EtOAc for different times (A). The plot of percentage of γ-CsPbI_3_ against the exposure time in EtOAc from nano-impact and XRD (B). The kinetic curves and their fitted curves obtained based on the JMAK model (C). Linear fitting of the experimental data using the logarithmic JMAK relation (D).

Next, XRD was further *ex situ* performed to investigate the phase transition kinetics of QDs during purification. Fig. 6A presents the XRD patterns of CsPbI_3_ QDs exposed to EtOAc for different times. It is observed that the diffraction intensity corresponding to the δ-CsPbI_3_ phase gradually increases with EtOAc treatment time from 0 to 10 min. In contrast, the crystal plane corresponding to γ-CsPbI_3_ decreases until it disappears completely. This proves that the phase transition of CsPbI_3_ QDs deepens with mixing time with EtOAc. The quantitative analysis of the phase evolution is summarized in Section 5 in the SI. The change from cubic structures to black dots in morphology is also confirmed by TEM analysis (Fig. S9), which is consistent with our previous report.^20^

The kinetics of phase transition determined from both nano-impact electrochemistry in 1 mM and 0 mM electrolyte and from XRD were then analyzed by plotting the remaining percentage of γ-CsPbI_3_ against time. Given that reduction of δ-CsPbI_3_ occurs at −1.3 V at 1 mM electrolyte (Fig. 4C) and at −1.4 V in 0 mM electrolyte (Fig. S8C), the percentage of γ-CsPbI_3_ in 1 mM electrolyte was calculated using the following formula: 
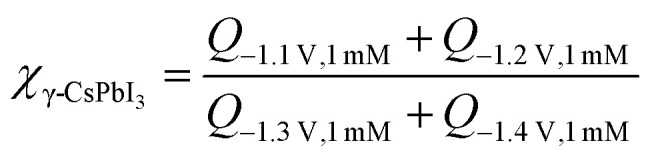
 and under electrolyte-free conditions using 
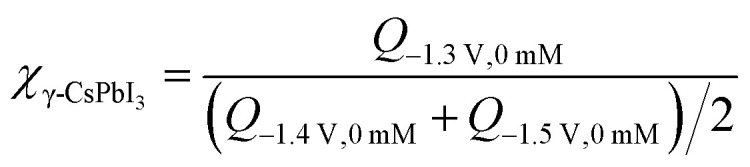
. *Q*_0 mM_ was found to be slightly lower than *Q*_1 mM_ from 0 to 5 min (Fig. S8), with about 0.46 fC lower at the reduction potential of δ-CsPbI_3_; this is likely due to the little inevitable aggregation in 1 mM electrolyte or the incomplete reduction in the absence of electrolyte. Within 5 min, the percentage of γ-CsPbI_3_ decreased from 96% to 36% in 1 mM electrolyte and from 96% to 44% in 0 mM electrolyte. In addition, after 5 min, *Q*_−1.4 V,0 mM_ and *Q*_−1.5 V,0 mM_ were much lower than *Q*_−1.3 V,1 mM_ and *Q*_−1.4 V,1 mM_, indicating that in 1 mM electrolyte, the CsPbI_3_ QDs were more prone to aggregation. Note that no spikes were detected at potentials below −1.3 V after 7 min (Fig. S7) for the electrolyte-free system, which is likely due to the insufficient γ-phase content from single QDs. As shown in Fig. 6B, the γ-phase fraction decreased over time, consistent with the kinetic trend observed from XRD. Such consistency provides evidence that the electrochemical measurement does not induce or alter the intrinsic phase transition process, but instead accurately reflects the purification-driven phase evolution of CsPbI_3_ QDs.

To further investigate the kinetic mechanism, the JMAK model was applied to fit the kinetic curves obtained from nano-impact electrochemistry at 1 mM electrolyte and from *ex situ* XRD: *x* = *e*^−(*kt*)*n*^ (Fig. 6C). By rearranging the above equation and taking the natural logarithm twice, the JMAK equation can be expressed as ln(−ln(*x*)) = *n* ln(*t*) + ln(*k*) to extract *k* and *n*. In this model, *x* represents the fraction of γ-phase, *t* is time, *k* is the effective rate constant which is temperature-dependent, and *n* is the Avrami constant (shape factor). Both *k* and *n* reflect contributions from nucleation and growth in multiple dimensions. *n* characterizes the nucleation rate, the growth dimension, and the rate-determining step of phase transition,^27^ expressed as *n* = *a* + *bc*, where *a* reflects the nucleation rate (*a* = 0 for a heterogeneous nucleation rate, 0 < *a* < 1 for a decreasing nucleation rate, *a* = 1 for homogeneous nucleation, and *a* > 1 for an increasing nucleation rate), *b* represents growth dimensionality (*b* = 1, 2, or 3 for 1D, 2D, or 3D growth), and *c* corresponds to the growth mode (*c* = 1 for surface-controlled growth; *c* = 1/2 for diffusion-controlled growth). The experimental kinetic curves were fitted to the linearized JMAK relation, and the Avrami exponent *n* was extracted directly from the slope of the fitted line, as shown in Fig. 6D. The Avrami exponent *n* values were approximately 1.14 ± 0.04 based on the nano-impact kinetics and 1.3 ± 0.2 from the *ex situ* XRD data, with corresponding *R*^2^ values of 0.987 and 0.907, respectively. The substantially higher *R*^2^ value for the nano-impact data indicates improved fitting of the transition kinetics compared with ensemble-averaged XRD analysis, likely due to the ability of nano-impact measurements to directly capture individual QDs and avoid averaging over heterogeneous populations. For CsPbI_3_ QDs, surface defects such as iodide vacancies have been reported as nucleation sites for δ-CsPbI_3_.^28^ Accordingly, the phase transition is assumed to proceed predominantly through heterogeneous surface nucleation, for which the value of *a* in this case is expected to approach 0. In addition, due to the small dimensions of QDs with high surface-to-volume ratios, transformation is governed not by volumetric growth within an extended lattice, but by the migration of a confined reaction front constrained by particle boundaries and surface chemistry.^29^ Therefore, the near-unity exponent is most consistent with heterogeneous nucleation at energetically favourable surface or defect sites, followed by low-dimensional interface-limited propagation of the δ-phase across the strongly confined nanocrystal.^30,31^ Note that under these strong confinement conditions, the Avrami exponent is an effective kinetic descriptor rather than a strict measure of geometric dimensionality. These results demonstrate that the nano-impact method provides more accurate and precise kinetic parameters than the ensemble-averaged *ex situ* XRD and is a highly potential approach for investigating the phase transition kinetics of QDs.

## Conclusions

In summary, an effective *in situ* platform based on nano-impact electrochemistry was developed for probing the phase transition kinetics of CsPbI_3_ QDs at the single-particle level. Electrolyte concentration was first optimized *via ex situ* CV on both GC and CF electrodes to ensure reliable electrochemical detection. Subsequently, nano-impact experiments were conducted at low electrolyte concentration using CA to *ex situ* determine the phase transition level of purified QDs. Building upon this, *in situ* nano-impact measurements employing both CV and CA enabled real-time tracking of the γ-to-δ phase transition at the single-particle level during solvent-induced purification. Kinetic analysis using the JMAK model suggests a transformation pathway governed by heterogeneous nucleation followed by low-dimensional interfacial propagation under strong nanoscale confinement. Compared with ensemble-averaged *ex situ* XRD, the nano-impact approach provides more accurate and precise kinetic parameters and captures the intrinsic heterogeneity of individual nanoparticles. These results highlight the critical role of surface nucleation sites and confined interfacial reconstruction in governing phase stability during purification, providing a rational basis for optimizing purification protocols. Although demonstrated here using the γ-to-δ phase transition of CsPbI_3_ QDs, the nano-impact approach provides a general framework for probing phase evolution in unstable nanomaterials with distinguishable phase-dependent electrochemical behaviors. This work advances the fundamental understanding of perovskite QD phase transitions and provides valuable guidance for the rational design of more stable QDs for optoelectronic and photovoltaic applications.

## Author contributions

Xiaolin Zhang and Yu Tian: investigation, data curation, formal analysis and writing. Yuanhang Zhu: investigation and data curation. Nanlin Zhang: resources and writing – review and editing. Chin-Wen Chen: resources and supervision. Xiuting Li: conceptualization, resources, supervision, and writing – review and editing.

## Conflicts of interest

There are no conflicts to declare.

## Supplementary Material

SC-OLF-D6SC04719J-s001

## Data Availability

The data supporting this article have been included as part of the supplementary information (SI). Supplementary information: chemicals and experimental methods, Fig. S1–S9 and Tables S1 and S2. See DOI: https://doi.org/10.1039/d6sc04719j.
