# Supplementary material for: In situ probing of phase transition kinetics of single CsPbI3 perovskite quantum dots via nano-impact electrochemistry
Source: Chem Sci. 2026 Aug 6;17(38):18444–53. doi: 10.1039/d6sc04719j (PMC13472196; doi:10.1039/d6sc04719j)
Supplement: Supplementary file 1 [file SC-017-D6SC04719J-s001.pdf]

Supplementary Information (SI) for Chemical Science.
This journal is © The Royal Society of Chemistry 2026

Supplementary Information for

In situ probing of phase transition kinetics of single $CsPbI_3$ perovskite quantum dots *via* nano-impact electrochemistry

Xiaolin Zhang,† [a] Yu Tian,† [a] Yuanhang Zhu, [a] Nanlin Zhang, [b] Chin-Wen Chen, [c] and Xiuting Li*[a]

[a] Institute for Advanced Study, Shenzhen University, Shenzhen 518060, China

[b] YangMing Quantum Technology LTD., Shenzhen 518000, China

[c] Department of Molecular Science and Engineering, National Taipei University of Technology, Taipei 10608, Taiwan

Corresponding Author

*Correspondence to: xiuting.li@szu.edu.cn.

Section 1:

Experimental Section

(1) Chemicals

Cesium carbonate (Cs_2CO_3), oleic acid, octadecene (ODE) and lead iodide (PbI_2) were provided by Sigma-Aldrich. Oleylamine and tetrabutylammonium hexafluorophosphate (Bu_4NPF_6) were purchased from Macklin. Hexane and ethyl acetate (AR, 99.5%, $H_2O \leqslant 0.1\%$, $CH_3OH \leqslant 0.1\%$, $CH_3CH_2OH \leqslant 0.1\%$ and $CH_3COOCH_3 \leqslant 0.1\%$) were bought from (Chronchem Chengdu). Argon (Ar) was supplied from (ShenTe Gas Shenzhen). Dichloromethane (DCM) was acquired from (Damas-beta Shanghai).

(2) Preparation of cesium oleate solution

0.5 g Cs_2CO_3 powder, 2 mL oleic acid and 50 mL ODE were added into a three-necked flask and heated to 90°C. After vacuumed for 1 hour, the flask was filled with nitrogen and heated to 120°C until all Cs_2CO_3 reacts with oleic acid to form cesium oleate. The ODE solution of cesium oleate is stored at room temperature under nitrogen. The cesium oleate solution needs to be preheated to 70°C under vacuum condition for half an hour before use.

(3) Synthesis of $CsPbI_3$ QDs

50 mL ODE and 1 g PbI_2 were added into a three-necked flask, stirred, vacuumed and heated. After the temperature reached 90°C, the reactants were left to set for 1 hour and then 5 mL oleic acid and 5 mL oleylamine were injected into them with a continuous supply of nitrogen. After injection, the flask was vacuumed for 2 hours at 90°C and heated to 160°C while nitrogen was filled. Once the temperature was reached, 8 mL cesium oleate (0.0625 M) prepared in the previous step, which was preheated to 70°C and vacuumed, was rapidly injected into the flask (below the liquid level). After 5 seconds, the flask was immediately placed in water bath and cooled to room temperature[1, 2].

(4) Purification of $CsPbI_3$ QDs

First, the above reaction solution was evenly divided into four centrifuge tubes containing methyl acetate ($\frac{V_{\text{reaction solution}}}{V_{\text{methyl acetate}}}$ = 1 : 3). After being shaken evenly, the four mixtures were centrifuged for 5 min at a speed of 8000 RPM. Then the supernatant of the centrifuge tubes was poured out and 3 mL

hexane was quickly added to each centrifuge tube to disperse the precipitated quantum dots. The sample in one of the centrifuge tubes was used as unpurified $CsPbI_3$ QDs in subsequent experiments. 3 mL and 39 mL ethyl acetate were added into other two centrifuge tubes respectively. After centrifugation for 5 min at 8000 RPM and removal of supernatant, the precipitates were quickly dispersed in 3 mL hexane. The two samples were then left in the freezer for two hours and centrifuged at 4,000 RPM for 3 min. The supernatants were collected and the $CsPbI_3$ QDs (1 : 1) and $CsPbI_3$ QDs (1 : 13) were obtained respectively. In addition, $CsPbI_3$ QDs (1 : 13 × 2) were obtained by performing the purification with 39 mL EtOAc twice[3, 4].

(5) Ex-situ electrochemical measurement of the $CsPbI_3$ QDs

Glassy carbon (GC) electrode (of diameter ca. 3.0 mm) and carbon fiber (CF) electrode (of diameter ca. 7.0 μm) were polished with three grades of aluminium powder (1.0, 0.3 and 0.05 μm), and then cleaned with water and dried with Ar. The purified $CsPbI_3$ QDs suspension was dropcast on the GC electrode by micro-syringe and left to dry under air atmosphere. In addition, the mass of the perovskite coated on the electrode surface was controlled at about 30 μg. The CF electrode was placed vertically into the purified sample and hold for 5 s.

Cyclic voltammetry (CV) was then conducted in a Faraday cage with an EC-Lab potentiostat using three electrode system at 298 K. The modified GC/CF electrode was used as the working electrode, with an Ag/AgCl electrode in 3 M KCl solution as the reference electrode and a platinum cylinder as the counter electrode. 15 mL Bu_4NPF_6 dichloromethane solution (100 mM, 10 mM, 1 mM and 0 mM) was degassed with Ar for 20 min, and atmosphere of Ar was maintained during the experiment.

Chronoamperometry (CA) were performed on Bu_4NPF_6 dichloromethane solution (1 mM and 0 mM) containing 6 μL purified sample ($CsPbI_3$ QDs (1 : 1), $CsPbI_3$ QDs (1 : 13) and $CsPbI_3$ QDs (1 : 13 × 2)) in the same experimental system as above except that the bare CF electrode was used as the working electrode.

(6) In-situ electrochemical measurement of the $CsPbI_3$ QDs

Measurements were carried out in the equipment of the above CA experiment. Both CV and CA was performed on Bu_4NPF_6 dichloromethane solution (1 mM and 0 mM) containing 20 μL EtOAc and 6

μL unpurified sample for different aging time.

Section 2:

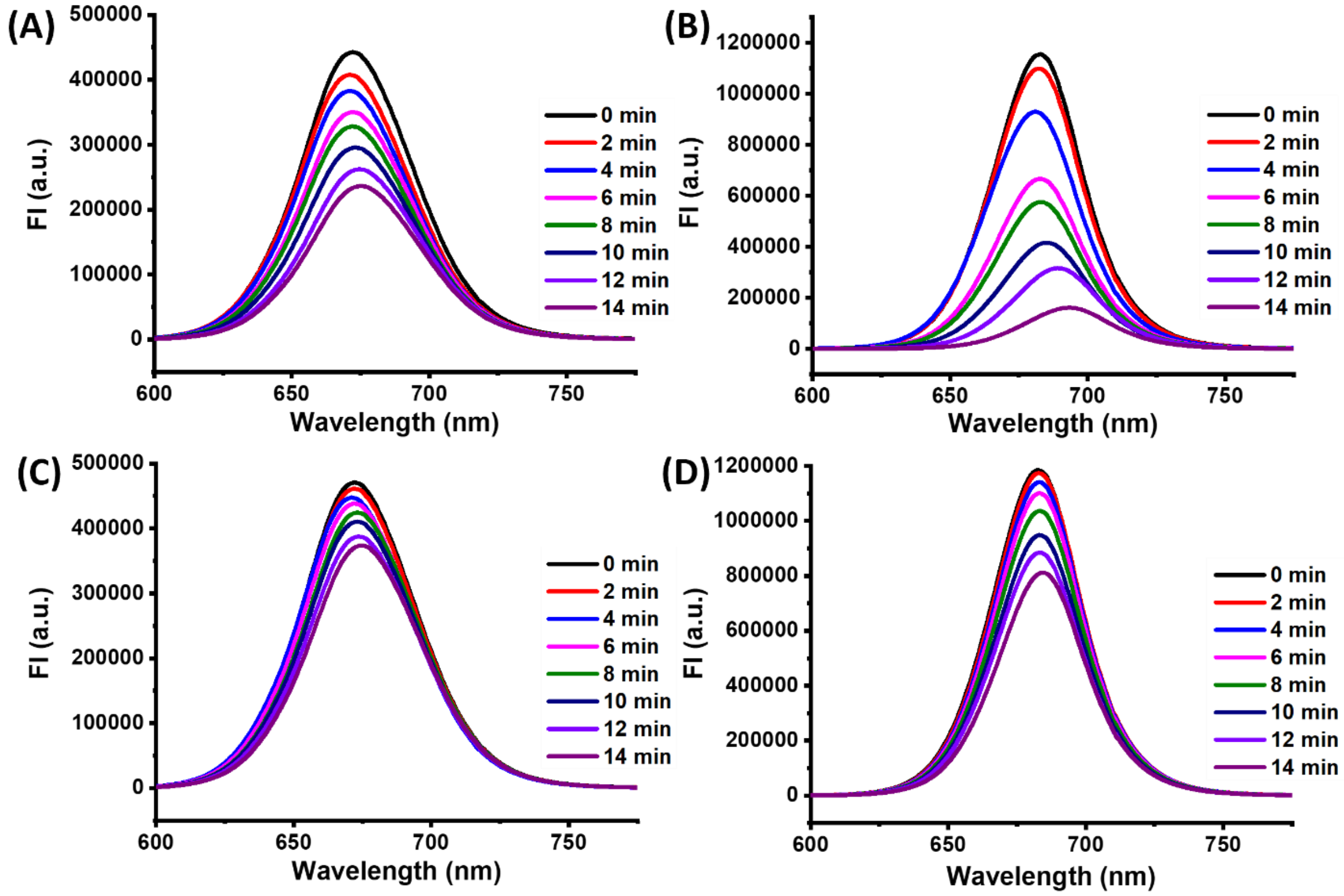

Fig. S1 PL spectra of unpurified $CsPbI_3$ QDs (A) and $CsPbI_3$ QDs (1 : 13) (B) at electrolyte concentrations of 100 mM; PL spectra of unpurified $CsPbI_3$ QDs (C) and $CsPbI_3$ QDs (1 : 13) (D) at electrolyte concentrations of 1 mM.

Section 3:

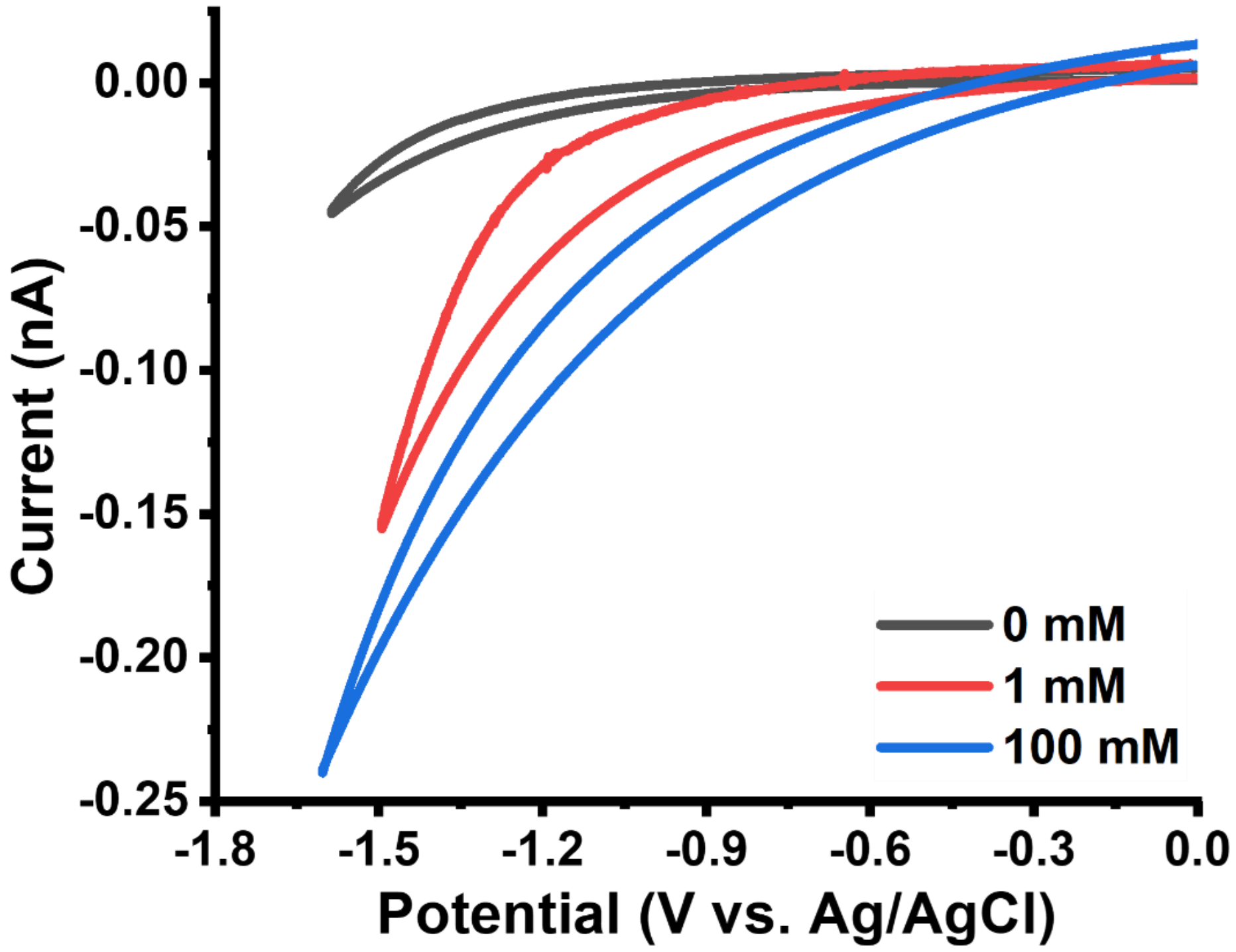

Fig. S2 CV of bare CF electrode in 0 mM (black), 1 mM (red) and 100 mM (blue) electrolyte.

Section 4:

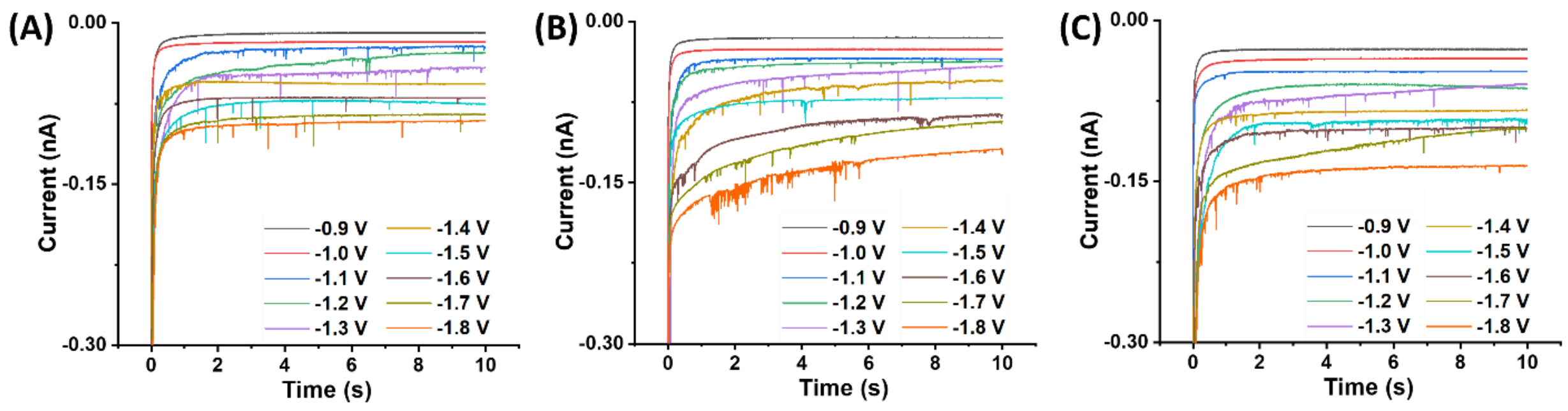

Fig. S3 Impact signals generated by $CsPbI_3$ QDs (1 : 1) (A), $CsPbI_3$ QDs (1 : 13) (B) and $CsPbI_3$ QDs (1 : 13 × 2) (C) at different potentials in the presence of 1 mM electrolyte.

Section 5:

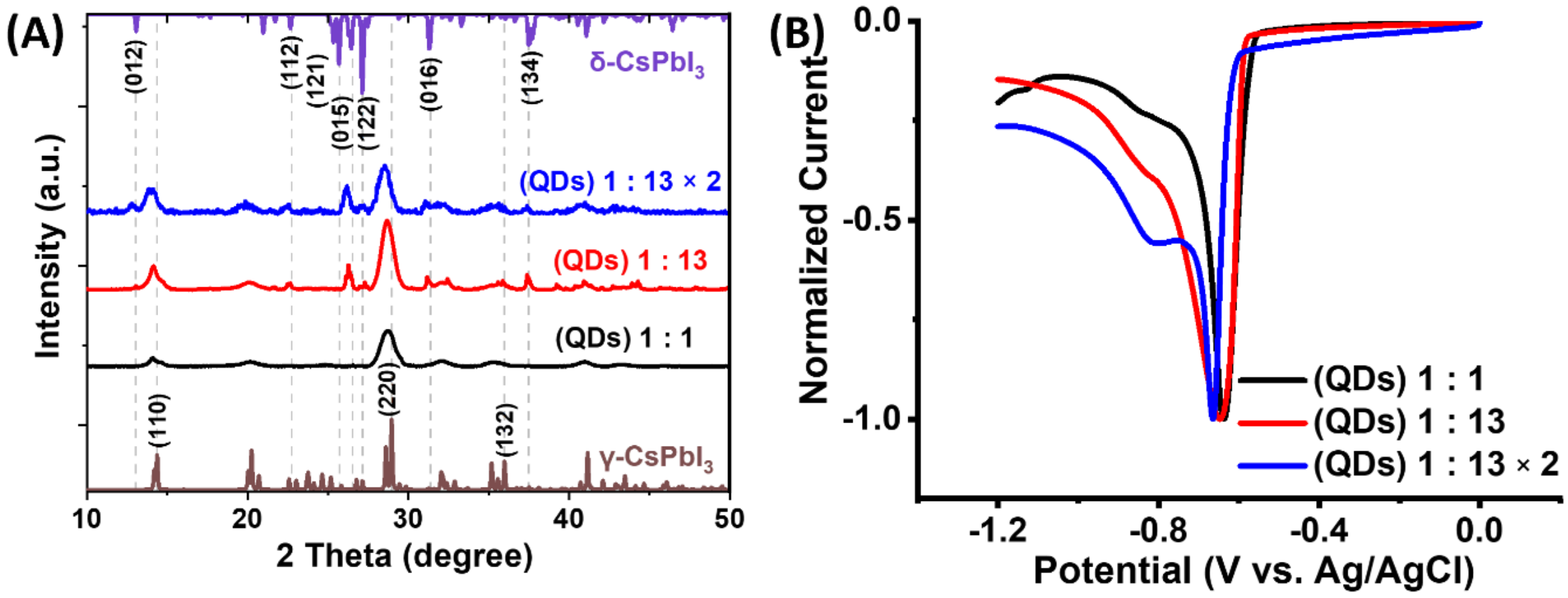

Fig. S4 XRD (A) of the $CsPbI_3$ QDs (1 : 1) (black), $CsPbI_3$ QDs (1 : 13) (red) and $CsPbI_3$ QDs (1 : 13 × 2) (blue), the standard γ-phase $CsPbI_3$ (purple) and δ-phase (brown) $CsPbI_3$ obtained from fitting the relevant data in the literature[5] and the NO.18-0376 standard card respectively; CV of the GC electrode dropcast with the $CsPbI_3$ QD samples (B).

Section 6:

Table S1. XRD peak intensity of two phases and percentage of γ-$CsPbI_3$ of different samples.

**$CsPbI_3$ QDs samples**	**Average peak intensity ((121), (015), and (122) planes) of δ-$CsPbI_3$**	**The (220) plane peak intensity of γ-$CsPbI_3$**	**Percentage of γ-$CsPbI_3$**
$CsPbI_3$ QDs (1 : 1)	4.05	218.45	97.08%
$CsPbI_3$ QDs (1 : 13)	93.55	421.98	73.56%
$CsPbI_3$ QDs (1 : 13 × 2)	91.58	291.38	66.24%

In the ex-situ XRD measurements monitoring the phase transformation kinetics over time, the percentage of γ-$CsPbI_3$ was estimated by the following formula:

$$\chi_{\gamma-\mathrm{CsPbI_3}} = \frac{I_\gamma / I_{\gamma,0\mathrm{min}}}{I_\gamma / I_{\gamma,0\mathrm{min}} + I_\delta / I_{\delta,10\mathrm{min}}}$$

I_γ represents the intensity of the highest (predominant) diffraction peak of γ-$CsPbI_3$ corresponding to the (220) plane. I_δ is calculated as the average intensity of the three most characteristic and diffraction peaks, associated with the (121), (015), and (122) lattice planes since they are continuous and difficult to be separated. In the equation, I_γ and I_δ are normalized by $I_{\gamma,0\mathrm{min}}$ and $I_{\delta,10\mathrm{min}}$ to eliminate differences in peak response. Subsequent experiments revealed that $CsPbI_3$ QDs exist entirely in the γ phase at 0 min and are fully transformed into the δ phase after 10 min of mixing with a polar solvent. $I_{\gamma,0\mathrm{min}}$ and $I_{\delta,10\mathrm{min}}$ correspond to these reference intensities.

Section 7:

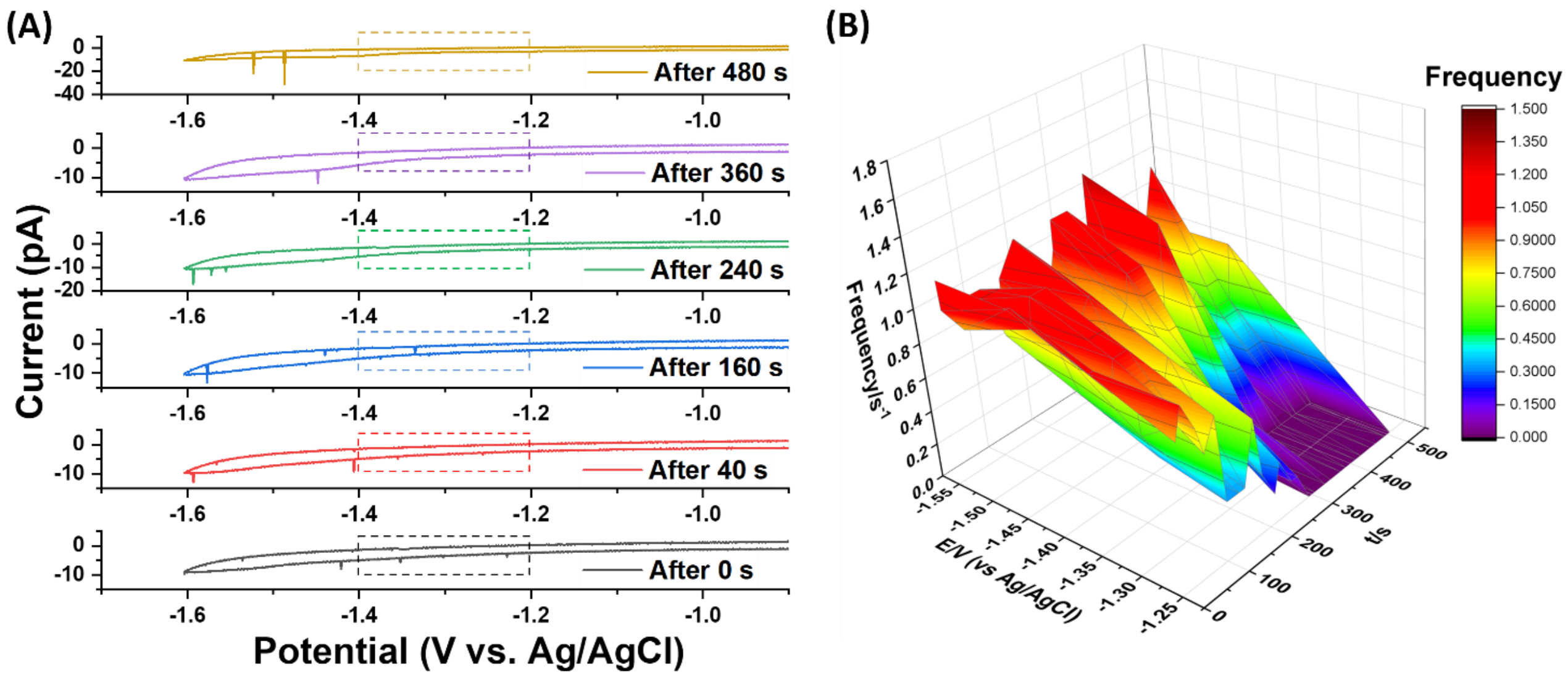

Fig. S5. Cyclic voltammograms of $CsPbI_3$ QDs in the absence of extra electrolyte (A). 3D maps plotting impact frequency against time and potential (B).

Section 8:

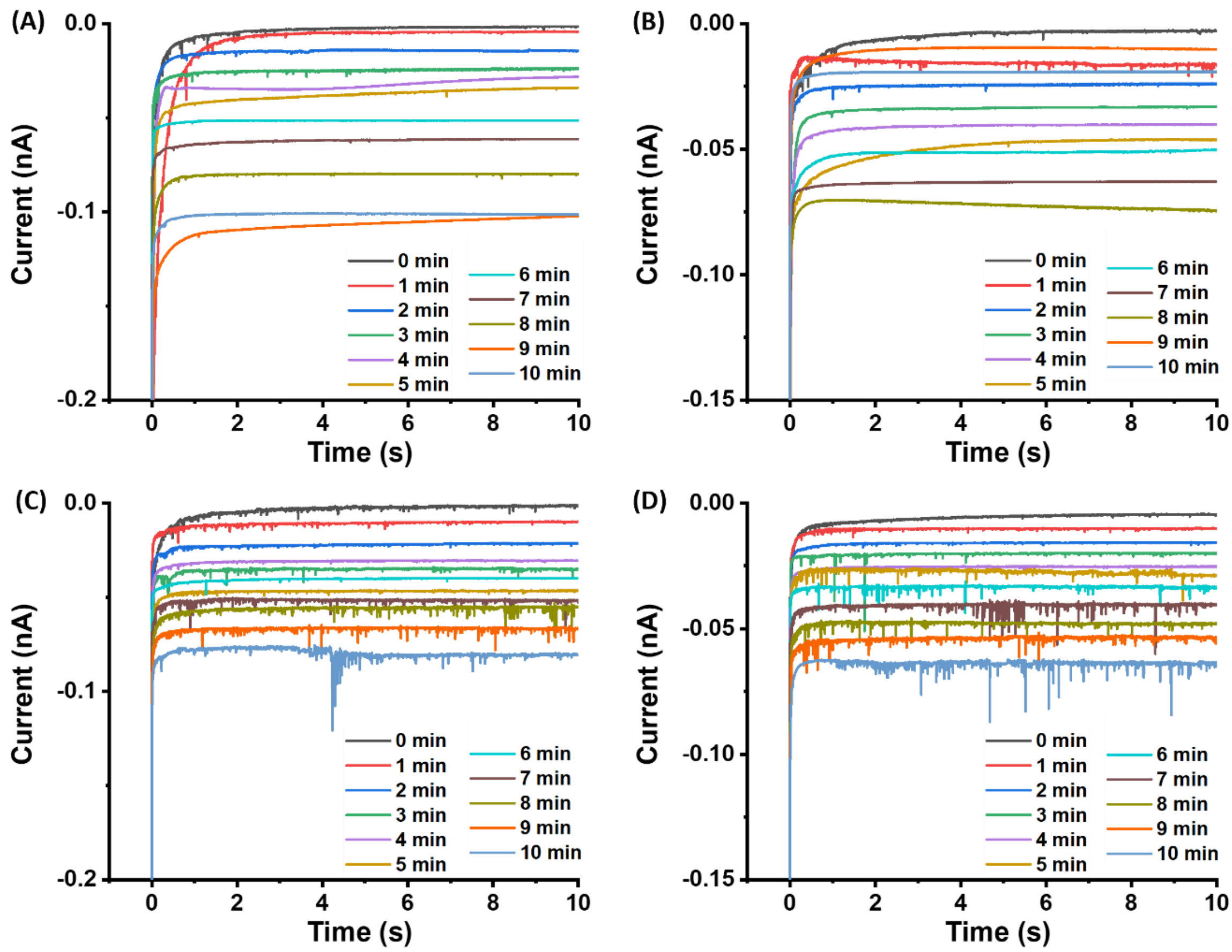

Fig. S6. CA of the $CsPbI_3$ QDs exposed to EtOAc at −1.1 V (A), −1.2 V (B), −1.3 V (C) and −1.4 V (D) in 1 mM electrolyte.

Section 9:

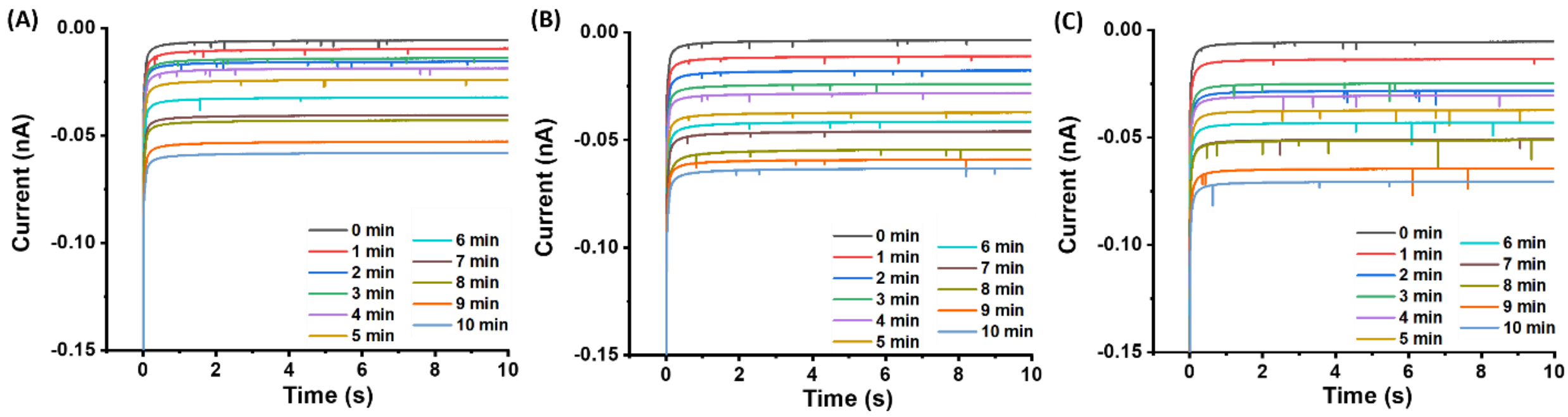

Fig. S7. CA of the $CsPbI_3$ QDs exposed to EtOAc at −1.3 V (A), −1.4 V (B) and −1.5 V (C) without extra electrolyte.

Section 10:

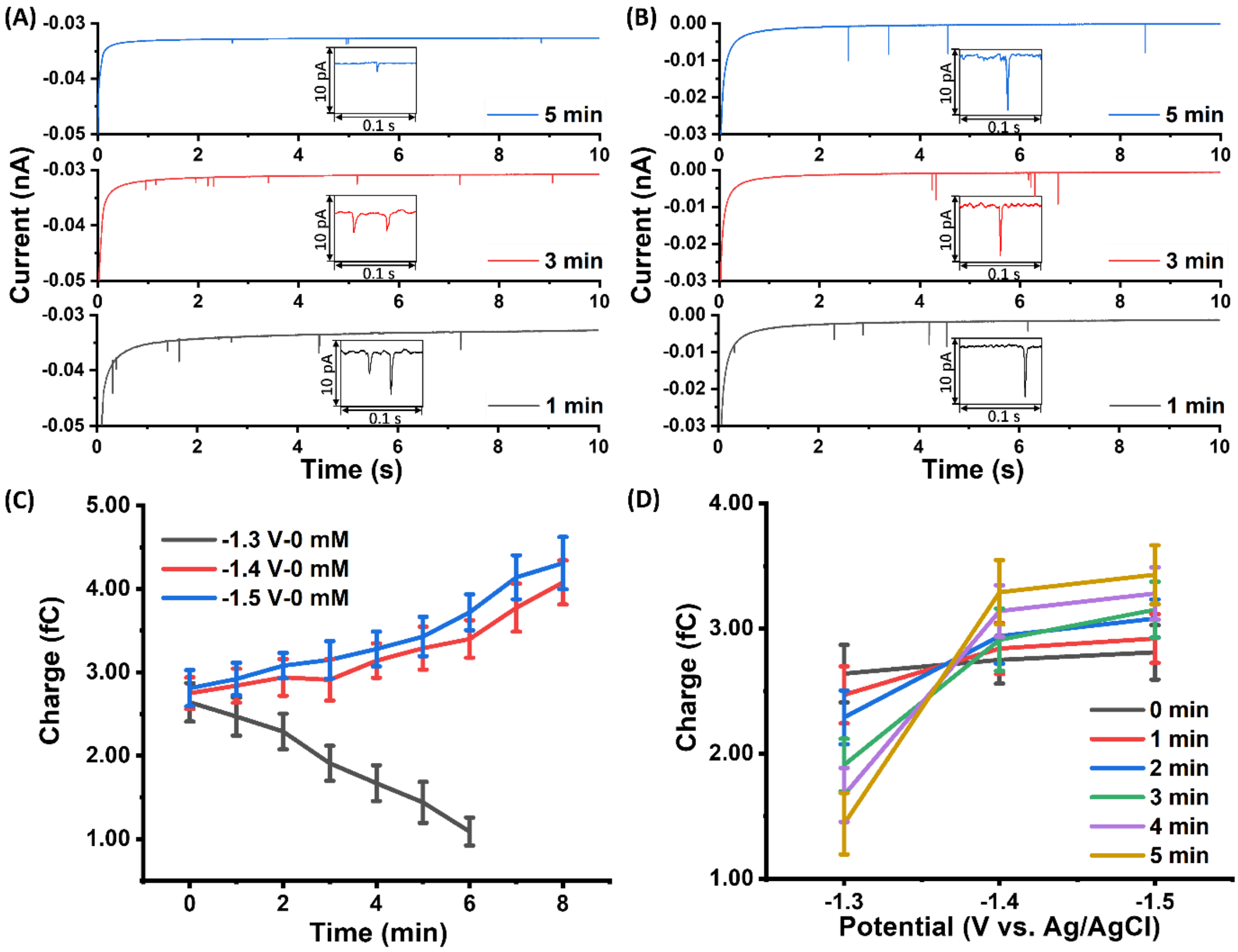

Fig. S8. CA of $CsPbI_3$ QDs exposed to EtOAc for different time at −1.3 V (A) and −1.5 V (B) without extra electrolyte; Charge statistics of spikes obtained under different potentials and time (C, D).

Section 11:

Table S2. XRD peak intensity of two phases and percentage of γ-$CsPbI_3$ of unpurified sample exposed to EtOAc for different time.

**Exposure time to EtOAc**	**Average peak intensity ((121), (015), and (122) planes) of δ-$CsPbI_3$**	**The (220) plane peak intensity of γ-$CsPbI_3$**	**Percentage of γ-$CsPbI_3$**
0 min	779	25872	95.35%
1 min	2342	26535	87.48%
3 min	10558	26433	60.69%
5 min	10710	12246	41.35%
7 min	13190	11936	35.82%
9 min	15461	5737	18.62%
10 min	15956	448	1.70%

Section 12:

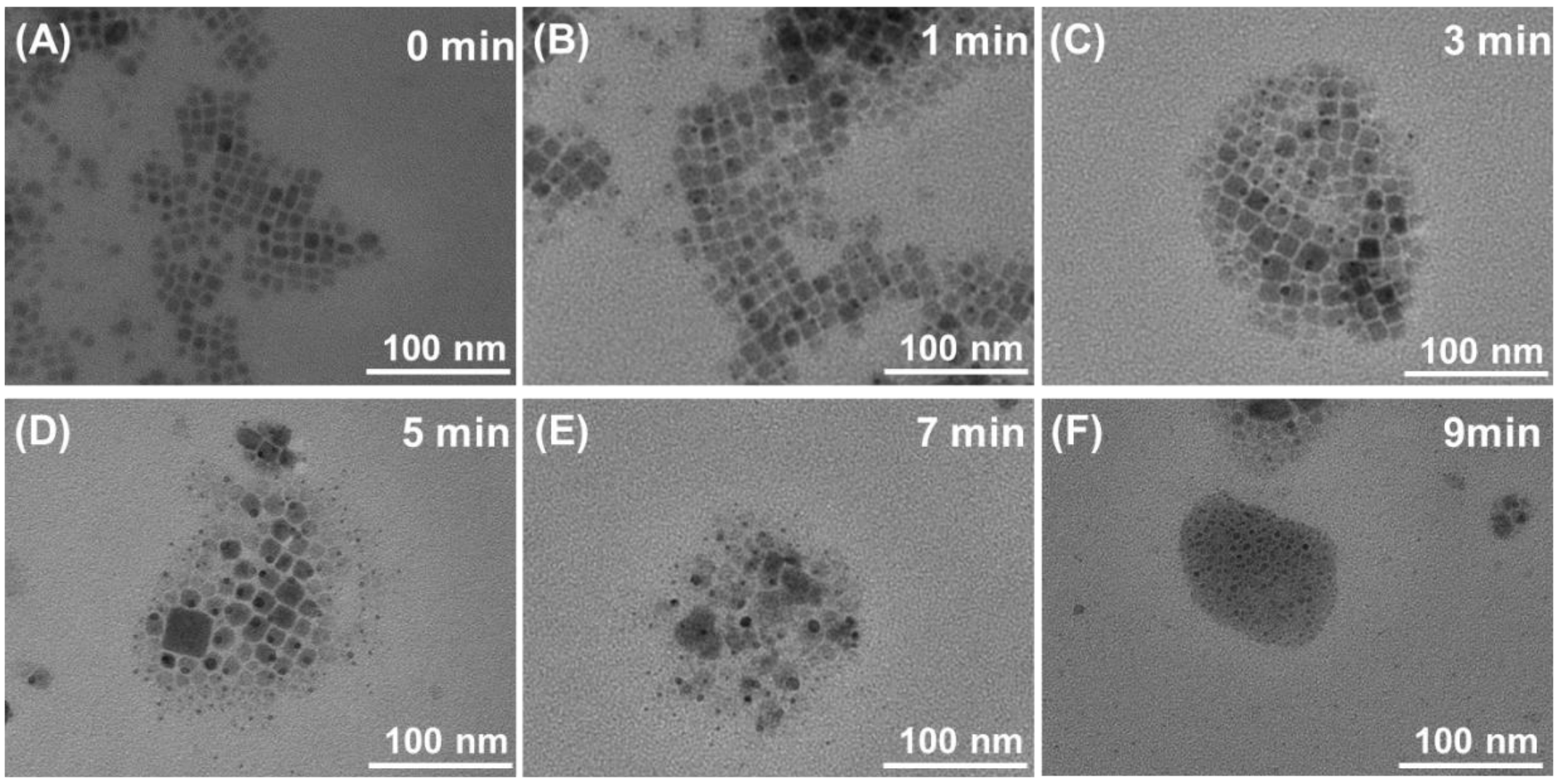

Fig. S9. TEM of $CsPbI_3$ QDs exposed to EtOAc for 0 min (A), 1 min (B), 3 min (C), 5 min (D), 7 min (E) and 9 min (F).

References:

1 L. Protesescu, S. Yakunin, M. I. Bodnarchuk, F. Krieg, R. Caputo, C. H. Hendon, R. X. Yang, A. Walsh and M. V. Kovalenko, Nanocrystals of cesium lead halide perovskites ($CsPbX_3$, X = Cl, Br, and I): novel optoelectronic materials showing bright emission with wide color gamut, *Nano Lett.*, 2015, **15**, 3692–3696.

2 X. Zhang, B. Xu, J. Zhang, Y. Gao, Y. Zheng, K. Wang and X. W. Sun, All-inorganic perovskite nanocrystals for high-efficiency light emitting diodes: dual-phase $CsPbBr_3$-$CsPb_2Br_5$ composites, *Adv. Funct. Mater.*, 2016, **26**, 4595–4600.

3 A. Swarnkar, A. R. Marshall, E. M. Sanehira, B. D. Chernomordik, D. T. Moore, J. A. Christians, T. Chakrabarti and J. M. Luther, Quantum dot–induced phase stabilization of α-$CsPbI_3$ perovskite for high-efficiency photovoltaics, *Science*, 2016, **354**, 92–95.

4 J. Shi, F. Li, Y. Jin, C. Liu, B. Cohen-Kleinstein, S. Yuan, Y. Li, Z. K. Wang, J. Yuan and W. Ma, In situ ligand bonding management of $CsPbI_3$ perovskite quantum dots enables high-performance photovoltaics and red light-emitting diodes, *Angew. Chem. Int. Ed.*, 2020, **59**, 22230–22237.

5 R. J. Sutton, M. R. Filip, A. A. Haghighirad, N. Sakai, B. Wenger, F. Giustino and H. J. Snaith, Cubic or orthorhombic? Revealing the crystal structure of metastable black-phase $CsPbI_3$ by theory and experiment, *ACS Energy Lett.*, 2018, **3**, 1787–1794.
